# Author Index

**DOI:** 10.1038/sj.bjc.6601058

**Published:** 2003-06-27


					Author Index
Abdel-Rahman AT S19 (5.6)
Ablin RJ S46 (P86)
Abraham J S57 (P129), S70 (P181),
S71 (P183)
Adamaki M S54 (P115), S54 (P118)
Adams L S71 (P184)
Adlard JW S55 (P119)
Agrawal RK S9 (CT1)
A'Hern R S58 (P132)
Ahmed S S18 (5.2), S34 (P38)
Ah-See M-LW S41 (P66), S44 (P77)
Airley R S52 (P109)
Airley RE S12 (1.6)
Aitken M S18 (5.2)
Al Mufti R S19 (5.5)
Ali K S23 (7.6)
Ali S S4 (S16)
Allum W S9 (CT3)
Almushatat A S61 (P146)
Al-Nafussi A S57 (P128),
S71 (P186)
Al-Rawi MAA S14 (3.2)
Amarnath SMP S40 (P62)
Andersen J S19 (5.4)
Anderson C S44 (P76)
Anderson CJ S39 (P59)
Anderson RJ S40 (P61)
Anderson C S65 (P162)
Anderton MJ S60 (P139)
Andreopoulou E S22 (7.2)
Anitha B S58 (P131)
Ansell W S62 (P147)
Armstrong A S37 (P49)
Armstrong AC S65 (P161)
Armstrong JL S67 (P168)
Aronow BJ S67 (P167)
Ashcroft M S39 (P59)
Ashley S S20 (5.8), S22 (7.2)
Ashley SE S19 (5.3)
Atkinson J S69 (P175)
Awan A S49 (P98)
Babalghith AO S59 (P137)
Baig N S11 (1.2)
Baildam AD S45 (P80a)
Bailly C S1 (S3)
Balkwill F S57 (P130)
Banerjee SS S65 (P161)
Banerji U S41 (P67)
Banwell CM S59 (P136)
Barber P S33 (P35)
Barr L S45 (P80a)
Bass R S23 (7.4)
Bassi C S55 (P120)
Basu B S57 (P129), S71 (P183)
Basu S S30 (P24), S32 (P29),
S32 (P30)
Bateman AC S43 (P73)
Bathers S S9 (CT1)
Bavetsias V S21 (6.6)
Bayne M S38 (P53)
Begent R S65 (P159)
Begent RHJ S37 (P50), S37 (P51)
Beger HG S55 (P120)
Bell CL S51 (P103)
Bell SE S21 (6.5)
Belloti T S54 (P118)
Benepal T S56 (P126)
Beresford M S21 (6.4)
Berney DM S50 (P99), S64 (P156)
Berry JM S28 (P13)
Berry P S47 (P89)
Betts CD S63 (P151)
Bhardwa JM S50 (P99), S64 (P156),
S72 (P187)
Bhattacharyya M S62 (P147)
Bhattacharyya P S61 (P143)
Bibby MC S27 (P11), S27 (P12),
S30 (P24), S31 (P28)
Bilgi M S26 (P6)
Bilsland A S39 (P58)
Birch JM S52 (P108)
Birchall MA S70 (P182)
Birtle AJ S61 (P145)
Biswas S S57 (P129), S71 (P183)
Blackett AD S35 (P41)
Blackledge G S7 (S26)
Blaydes JP S29 (P20)
Bliss JM S69 (P176)
Bloodworth LC S51 (P104)
Board J S54 (P116)
Boddy AV S41 (P68), S53 (P111),
S55 (P122), S67 (P168)
Boden RW S37 (P50)
Boehler F S64 (P157), S64 (P158)
Bofill M S22 (6.7)
Bonner SE S19 (5.5)
Bonshek R S65 (P161)
Bosanquet N S21 (6.5)
Boulos N S18 (4.7)
Boxer GM S37 (P50), S37 (P51)
Boyd M S11 (1.2), S63 (P152)
Boyne J S17 (4.5)
Bradley RG S39 (P57)
Bradshaw TD S28 (P13)
Brakenhoff R S23 (7.6)
Braybrooke J S57 (P130)
Breen M S50 (P100)
Breitfellner G S64 (P157),
S64 (P158)
Brenner MK S2 (S8)
Brenton J S57 (P129), S71 (P183)
Bridgewater CH S24 (7.7)
Brookman-Amissah N S49 (P95)
Brooks AC S28 (P16)
Broome J S15 (3.6)
Brown A S23 (7.6)
Brown E S23 (7.5), S57 (P128)
Brown G S20 (6.1)
Brown I S26 (P6), S59 (P138)
Brown JE S32 (P30), S67 (P170)
Brown JM S21 (6.5)
Brown SB S27 (P10)
Brown SJ S33 (P34)
Brown T S40 (P61)
Bryden AAG S26 (P5)
Bubnovskaya LN S27 (P9)
Buchler MW S55 (P120)
Buggy Y S18 (5.1), S48 (P94)
Buluwela L S4 (S16)
Bunton S S68 (P171)
Burchill S S17 (4.5)
Burchill SA S52 (P110)
Burcombe RJ S41 (P66), S44 (P77)
Burdall S S20 (5.7), S60 (P142)
Burke GAA S53 (P112), S54 (P117)
Burke MD S33 (P36)
Buscombe J S65 (P159)
Bustin SA S19 (5.5)
Butler PC S27 (P11), S33 (P36)
Cairns D S40 (P61)
Calvert H S42 (P69), S7 (S27)
Cameron DA S58 (P134)
Campanella L S27 (P9)
Campbell MJ S59 (P136)
Campbell SJ S29 (P20)
Campisi J S5 (S17)
Candinas D S43 (P75)
Caplin M S65 (P159)
Carnell D S12 (1.5)
Carnell DM S34 (P37)
Carpenter R S19 (5.5),
S19 (5.6), S47 (P87)
Carr J S54 (P116)
Carr N S36 (P47)
Case MC S51 (P106)
Cassidy J S48 (P91)
Challen C S54 (P116)
Chalmers A S11 (1.1)
Chan C S2 (S6)
Chan HTC S36 (P45)
Chaplin T S46 (P85), S54 (P118)
Chapman J S65 (P161)
Chau I S20 (6.1)
Cheadle EJ S36 (P48), S37 (P49)
Chellam VG S58 (P131)
Cheng M-K S40 (P63)
Cheng Y S35 (P41)
Chester KA S37 (P50)
Chinewundoh FI S72 (P187)
Chinje EJ S30 (P21)
Chorny VA S43 (P75)
Chung Y-L S17 (4.3), S41 (P67)
Cladd HJ S41 (P66), S44 (P77)
Clark AM S11 (1.2)
Clark P S10 (CT6)
Clarke NW S49 (P97), S62 (P150),
S63 (P151)
Cleator S S19 (5.3)
Clifford SC S12 (2.1), S15 (3.4),
S50 (P101), S52 (P107),
S69 (P175)
Cole C S58 (P132)
Coleman R S25 (P1)
Coleman RE S18 (5.2),
S67 (P170)
Collie-Duguid E S45 (P82),
S48 (P92)
Collie-Duguid ESR S48 (P91)
Colston KW S13 (2.5), S14 (2.7),
S25 (P3), S25 (P4)
Cooke JC S15 (3.4)
Cookson JC S15 (3.3)
Coombes RC S4 (S16)
Cooper PA S27 (P11),
S29 (P19)
Corcoran F S37 (P51)
Cotter F S50 (P99)
Coulson JM S4 (S15)
Coulthard S S51 (P105)
Coulthard SA S51 (P106)
Cowan R S49 (P97)
Cowan RA S65 (P161)
Coxon JP S14 (2.7), S25 (P4)
Crabb SJ S65 (P160)
Cragg M S2 (S6), S26 (P7)
Cragg MS S36 (P45), S47 (P88)
Cranston D S45 (P81)
Crawshaw M S53 (P113)
Cross NA S26 (P5)
Cunningham D S20 (6.1),
S22 (6.8), S56 (P124),
S56 (P125), S58 (P133),
S72 (P188), S9 (CT3)
Cutress RI S59 (P135)
Dachs GU S30 (P22)
Daley FM S33 (P35), S34 (P37)
Dalgleish A S28 (P14), S44 (P76)
Dancey G S61 (P143), S62 (P147)
Darby AJ S37 (P52)
David W S65 (P161)
Davies C S44 (P77)
Davies MPA S43 (P74), S49 (P96)
Dawson LA S61 (P144)
Deakin DP S65 (P161)
Dearling J S37 (P51)
Debnath D S26 (P6)
Denny WA S2 (S5)
Deroide F S22 (6.7)
Dertinger S S64 (P158)
Dervernis C S55 (P120)
Di Salvo A S27 (P12)
Dibling BC S52 (P110)
Dolbear C S17 (4.4)
Double JA S27 (P12), S42 (P70)
Doughty SW S12 (1.7), S31 (P27),
S32 (P31)
Douglas S S58 (P134)
Douglas-Jones A S44 (P79),
S46 (P86)
Drbal AA S42 (P70)
Dredge K S28 (P14)
Drew PJ S40 (P62)
du Bois A S8 (S29)
Du Y S38 (P53), S38 (P54)
Duchesne F S68 (P174)
Duffy MJ S48 (P94)
Dunn JA S55 (P120), S9 (CT1)
DuPlessis D S15 (3.6), S42 (P71a)
Durrant C S22 (6.8)
Durrant L S38 (P56)
Durrant LG S39 (P57), S44 (P78)
Dyer CE S40 (P62)
Earl H S57 (P129), S70 (P181), S71
(P183)
Earl HM S9 (CT1)
Eaton C S25 (P1)
Eaton CL S26 (P5)
Eden OB S52 (P108)
Eden T S17 (4.6)
Eisen T S69 (P178)
Eiter H S64 (P157)
Ejlertsen B S19 (5.4)
El-Emir E S37 (P50)
El-Ghobashy AA S13 (2.4)
Elliott TJ S37 (P52)
Ellis S S67 (P170)
Ellison DW S12 (2.1), S50 (P101),
S52 (P107)
Elsworth AM S53 (P111)
Erenpreisa J S26 (P7)
Errington J S53 (P111), S41 (P68)
Estlin EJ S52 (P109)
Evans A S12 (1.6), S25 (P1)
Evans DGR S45 (P80a)
Evans G S66 (P164)
Evans WE S3 (S11)
Everard J S18 (5.2)
British Journal of Cancer (2003) 88(Suppl 1), S73 - S76
& 2003 Cancer Research UK All rights reserved 0007- 0920/03 $25.00
www.bjcancer.com
Everard M S58 (P132)
Everett SA S12 (1.7),
S33 (P35), S34 (P37)
Farmer PB S62 (P148)
Farnie G S34 (P38)
Fernandez-Cruz L S55 (P120)
Fernando I S57 (P127)
Ferry D S31 (P25)
Flannigan GM S32 (P30)
Fleming F S14 (3.1)
Fletcher D S54 (P118)
Fletcher-Monaghan AJ S40 (P64)
Folkes LK S30 (P23)
Ford H S23 (7.4)
Foster CS S43 (P72), S43 (P74)
Foster L S9 (CT1)
Fowler CG S72 (P187)
Fox MD S47 (P88)
Foxon R S49 (P95)
Francis R S23 (7.6)
Freeman A S61 (P145)
French R S2 (S6)
French RR S36 (P45)
Friess H S43 (P75), S55 (P120)
Fritzsche H S64 (P157)
Fullerton N S63 (P152)
Fussell HM S43 (P73)
Gabra H S57 (P128), S71 (P186),
S8 (S31)
Galbraith SM S12 (1.7)
Gallagher J S13 (2.3)
Gammerman A S54 (P118)
Ganesan TS S57 (P130)
Ganusevich II S27 (P9)
Geh I S20 (6.2)
Geh I S63 (P153)
Geldart T S36 (P47)
Gescher AJ S62 (P148)
Gibbens I S56 (P126), S58 (P132)
Gibbs D S21 (6.6)
Gibson BE S10 (CT5)
Gibson P S27 (P11)
Gilbertson RJ S69 (P175)
Gilham DE S36 (P48), S37 (P49),
S38 (P55)
Gill JH S30 (P24), S32 (P29),
S64 (P155)
Giridharan S S20 (6.2)
Given-Wilson R S13 (2.5)
Glaser A S53 (P113)
Glennie M S2 (S6), S36 (P47),
S38 (P54)
Glennie MJ S36 (P45), S36 (P46),
S47 (P88)
Gluzman DF S43 (P75)
Glynne-Jones R S21 (6.3), S21 (6.4)
Going JJ S40 (P64)
Goldie T S23 (7.4)
Goodhart IM S67 (P170)
Gordan S16 (4.1)
Gore M S56 (P126), S58 (P132)
Gornall P S16 (4.1)
Goulden N S4 (S13)
Goulden NJ S53 (P112), S54 (P117)
Grady M S52 (P109)
Graham CL S55 (P122)
Graham L S69 (P177)
Green M S21 (6.6)
Greenman J S40 (P62)
Greer ML S33 (P35)
Grieve RJ S9 (CT1)
Griffin MJ S41 (P68)
Griffiths J S27 (P10)
Griffiths JR S17 (4.3), S41 (P67)
Grindon CR S40 (P63)
Grundy R S16 (4.1)
Guest RD S36 (P48)
Guillou PJ S21 (6.5)
Gutcher S S67 (P170)
Guy M S13 (2.5)
Hajibagheri N S18 (4.8)
Hale J S41 (P68), S53 (P113)
Hall A S51 (P105)
Hall AG S51 (P104), S51 (P106)
Hall G S58 (P132)
Hall P S58 (P134)
Hamblin T S67 (P169)
Hambly R S66 (P163)
Hammal DM S51 (P103)
Hamzah AS S29 (P17)
Hanby A S20 (5.7)
Hancock BW S18 (5.2), S66 (P163)
Hankin SL S27 (P10)
Hanks S S16 (4.2)
Hann I S54 (P118)
Harland SJ S61 (P145)
Harley CB S5 (S18)
Harney J S11 (1.3), S41 (P65)
Harper-Wynne C S22 (6.8),
S22 (7.2)
Harper-Wynne CL S23 (7.4)
Harries M S58 (P132)
Harris AL S44 (P77), S45 (P81),
S57 (P130)
Harris JC S49 (P98)
Harrison C S38 (P53)
Harrison M S21 (6.4)
Hart IR S4 (S14)
Hartley A S20 (6.2), S57 (P127)
Hartley JM S37 (P51)
Harvey M S36 (P47)
Havranek EG S44 (P76)
Hawkins RE S36 (P48), S37 (P49),
S38 (P55)
Haylock B S15 (3.6)
Heald R S15 (3.3)
Healy S S71 (P184)
Hellmann K S42 (P71)
Helsby NA S31 (P25)
Hendry JH S62 (P150)
Hernan R S69 (P175)
Herod J S13 (2.4)
Herrington S S13 (2.4)
Hewitt S S12 (1.6)
Heys SD S26 (P6), S59 (P137),
S59 (P138)
Hickish T S22 (6.8)
Hill A S14 (3.1), S18 (5.1)
Hill ADK S14 (2.6), S48 (P94)
Hill M S20 (6.1), S22 (6.8),
S56 (P124)
Hill ME S56 (P125), S58 (P133)
Hiller L S9 (CT1)
Hine AV S48 (P93)
Hirst DG S32 (P32)
Ho GF S24 (7.7)
Hoare S S57 (P130)
Hoare SF S39 (P58)
Hochhauser D S65 (P159)
Hodges L S38 (P53)
Hogarth LA S51 (P104)
Holen I S25 (P1), S26 (P5)
Holme AL S26 (P8)
Honeychurch J S36 (P46),
S38 (P54)
Hooper R S23 (7.6)
Hoskin P S12 (1.5), S34 (P37)
Houlston RS S69 (P178)
Howell WM S43 (P73)
Howells LM S60 (P139)
Hubank M S54 (P118)
Hudson EA S60 (P139)
Hughes C S36 (P48)
Hughes CM S32 (P32)
Hughes D S36 (P45)
Hughes K S11 (1.2)
Hurle RA S62 (P149)
Hurley L S15 (3.3)
Husain E S19 (5.6)
Husband D S15 (3.6)
Hussain SA S63 (P153)
Hutton C S16 (4.1)
Ibbotson R S67 (P169)
Ibrahim K S24 (7.8)
Iddawela M S57 (P129)
Ijaz T S33 (P36)
Illidge T S26 (P7), S38 (P53),
S38 (P54)
Illidge TM S36 (P46), S65 (P160)
Imeson J S16 (4.1)
Innes H S43 (P74)
Innes J S13 (2.4)
Irlam J S36 (P48)
Irwin HL S37 (P50), S37 (P51)
Ivanov A S26 (P7)
Iveson T S22 (6.8)
Iwuagwu O S40 (P62)
Jackman A S56 (P126)
Jackman AL S21 (6.6)
Jackson DP S47 (P89)
Jada SR S29 (P17), S29 (P18)
Jagdev S S53 (P113)
James ND S35 (P44), S63 (P153)
Jankowski J S49 (P98)
Jayson G S13 (2.3)
Jenkins A S58 (P132)
Jenkins PJ S19 (5.5), S47 (P87)
Jenkins SA S62 (P149)
Jenkins TC S1 (S2), S28 (P15),
S29 (P19), S31 (P27),
S32 (P31), S40 (P61)
Jennings SA S28 (P15)
Jeyapalan JC S39 (P60)
Jiang M S34 (P40)
Jiang WG S14 (3.2), S44 (P79),
S45 (P80), S46 (P83),
S46 (P86), S60 (P140),
S60 (P141), S62 (P149)
Joel S S25 (P2), S66 (P165)
Joel SP S65 (P162)
John T S42 (P69)
Johnson L S69 (P176)
Johnson P S2 (S6), S23 (7.4),
S36 (P47), S38 (P53), S38 (P54)
Johnson PW S29 (P20)
Johnson PWM S15 (3.5),
S36 (P46), S37 (P52),
S59 (P135), S65 (P160)
Johnston P S11 (1.1)
Joiner M S11 (1.3)
Jones KP S16 (4.1)
Jones L S54 (P118)
Jones LK S18 (4.8), S54 (P115)
Jones R S28 (P14)
Joyce KA S15 (3.6), S42 (P71a)
Judson IR S41 (P67)
Kan KS S35 (P43)
Kanthou C S28 (P16)
Kassem HSh S49 (P97)
Katerinaki E S47 (P89), S66 (P163),
S66 (P164)
Katopodis O S72 (P188)
Kaur K S57 (P130)
Kay G S27 (P12)
Kaye S S58 (P132)
Kearns PR S53 (P112), S54 (P117)
Keith N S63 (P152)
Keith WN S39 (P58), S39 (P59),
S5 (S20), S40 (P64)
Kelsey A S16 (4.1), S52 (P109)
Kerr DJ S68 (P174)
Kerr K S45 (P82), S48 (P92),
S63 (P154)
Khal J S48 (P93)
Khan FK S18 (5.2)
King M S20 (6.2), S57 (P127)
King-Underwood L S16 (4.2)
Kirillova N S36 (P48)
Kirk D S63 (P152)
Kjaer M S19 (5.4)
Kleeff J S43 (P75)
Knox RJ S32 (P31)
Koller KE S53 (P111)
Kovelskaya AV S27 (P9)
Krishnasamy M S22 (7.1)
Kularatne B S66 (P163)
Kumar DM S24 (7.8)
Kumar M S50 (P99)
Kumar P S50 (P102)
Kumar S S50 (P102)
Kynaston HG S62 (P149)
Laban CA S19 (5.5)
Laban CA S47 (P87)
Lacaine F S55 (P120)
Lajis NH S29 (P17)
Lal KR S19 (5.3), S56 (P124)
Lamont JM S50 (P101)
Lane AN S1 (S1)
Lane DP S34 (P39), S35 (P42)
Lane J S60 (P140), S60 (P141)
Lane S S20 (5.7)
Langdon J S23 (7.6)
Langdon JA S69 (P175)
Langdon SP S13 (2.2)
Langkjer ST S19 (5.4)
Lanham S S67 (P169)
Laughton CA S28 (P13), S40 (P63)
Lavery K S23 (7.6)
Leach MO S17 (4.3), S41 (P66),
S41 (P67)
Learmonth T S71 (P184)
Leatherbarrow B S65 (P161)
Ledermann JA S9 (CT2)
Leslie M S51 (P106)
Levitin IYa S27 (P9)
Lewington V S38 (P53)
Lewis I S9 (CT4)
Lim KT S14 (2.6)
Limer J S20 (5.7)
Lind MJ S68 (P171)
Lindsey JC S12 (2.1), S52 (P107)
Lippit J S26 (P5)
Lister TA S65 (P162)
Little S S16 (4.2)
Liu W S25 (P2), S46 (P85), S65
(P162), S66 (P165)
Livingstone A S11 (1.2)
Lloveras B S22 (6.7)
Loadman PM S27 (P11), S27 (P12),
S29 (P19), S31 (P26),
S32 (P29), S32 (P30),
S64 (P155)
Lorigan P S66 (P163), S66 (P164)
Author Index
S74
British Journal of Cancer (2003) 88(Suppl 1), S73 - S76 & 2003 Cancer Research UK
Lorigan PC S47 (P89)
Lowe LC S13 (2.5), S25 (P3)
Lukyanchuk VV S43 (P75)
Lunec J S18 (4.7), S34 (P38),
S54 (P116)
lup Wong W S41 (P65)
Lusher ME S12 (2.1), S52 (P107)
Lyle J S27 (P12)
Lyn BE S23 (7.3)
Lynch CB S35 (P44)
Mackay A S49 (P95)
Mackean M S23 (7.5)
MacLeod K S13 (2.2)
MacNeil S S47 (P89), S66 (P164)
Maddineni SB S62 (P150),
S63 (P151)
Madhusudan S S57 (P130)
Madjd Z S44 (P78)
Maguire TM S48 (P94)
Maharaj L S65 (P162)
Mairs R S63 (P152)
Mairs RJ S11 (1.2)
Maitland NJ S61 (P144)
Makris A S21 (6.4), S41 (P66),
S44 (P77)
Malpas J S46 (P85)
Manikam SD S26 (P8)
Mansel RE S14 (3.2), S44 (P79),
S45 (P80), S46 (P86),
S60 (P140)
Mansi JL S13 (2.5), S14 (2.7)
Manson MM S60 (P139)
Maraveyas A S68 (P171)
Marcusson E S15 (3.5)
Margison GP S49 (P97), S62 (P150)
Marriott B S28 (P14)
Martin J S50 (P99)
Martin SG S11 (1.4)
Martin SW S32 (P29), S32 (P30),
S64 (P155)
Martin TA S60 (P141)
Massey A S20 (6.1), S56 (P124)
Masters JRM S61 (P145)
Matakidou A S69 (P178)
Mathews C S29 (P17)
Matthews CS S28 (P13)
Mawdsley S S21 (6.3)
Mayer A S70 (P181)
Mazhar D S4 (S16)
Mc Dermott E S14 (3.1),
S18 (5.1)
McArdle PA S61 (P146)
McCarthy HO S32 (P32)
McCarthy K S19 (5.5)
McConkey C S20 (6.2),
S57 (P127)
McCorroll A S29 (P17)
McDermott E S48 (P94)
McDermott EW S14 (2.6)
McDonald AC S55 (P121)
McDonald SL S59 (P137),
S59 (P138)
McErlane V S32 (P32)
McFadyen MCE S16 (3.8)
McGreal G S48 (P94)
McKeown SR S32 (P32)
McMahon L S55 (P121)
McManamy CS S50 (P101)
McMillan DC S61 (P146)
McNally RJQ S52 (P108)
McPhillips F S13 (2.2)
Mellon JK S62 (P148)
Melton R S55 (P122)
Melvin WT S16 (3.8)
Metheringham R S38 (P56)
Meyer T S65 (P159)
Michael A S16 (3.7)
Michels J S15 (3.5)
Middleton G S56 (P124)
Middleton H S16 (4.1)
Millard LJ S61 (P143)
Milner J S34 (P40)
Mincher DJ S27 (P12)
Mirnezami AH S29 (P20)
Mitchell C S16 (4.1)
Mitchell F S56 (P126)
Mitchell MP S54 (P115)
Mitra SS S20 (5.8)
Modi C S40 (P63)
Mohanamurali J S71 (P185)
Moir SE S59 (P137), S59 (P138)
Mller H S17 (4.4)
Monia B S13 (2.2)
Moody M S70 (P181)
Moores SL S28 (P15), S31 (P27),
S32 (P31)
Morgan DAL S11 (1.4)
Morgan J S39 (P57)
Morgan S S2 (S6)
Moss C S58 (P132)
Moss PA S35 (P44)
Moss RS S38 (P56)
Mouridsen HT S19 (5.4)
Mullen P S13 (2.2)
Mullerat J S22 (6.7)
Murphy DM S17 (4.4)
Murray G S45 (P82)
Murray GI S16 (3.8), S33 (P35),
S34 (P37), S48 (P91), S48 (P92)
Myers E S18 (5.1)
N'Dow J S46 (P84)
Nair TKG S58 (P131)
Napp V S69 (P177)
Nargund V S50 (P99), S64 (P156)
Nargund VH S72 (P187)
Nashed MS S34 (P39)
Naylor MA S12 (1.7)
Neat MJ S54 (P115)
Neidle S S5 (S19)
Neil E S71 (P184)
Neilson L S40 (P64)
Neoptolemos JP S55 (P120)
Neville-Webb H S68 (P171)
Neville-Webbe HL S25 (P1)
Newbold RF S18 (4.7)
Newell DR S55 (P122)
Ng S S53 (P114)
Nicholson M S22 (6.8)
Nicolaou A S42 (P70)
Nicolson M S63 (P154), S71 (P184)
Nicolson MC S45 (P82), S48 (P92)
Noble JL S65 (P161)
Nooij M S9 (CT4)
Norman A S20 (6.1), S22 (6.8)
Norman AR S56 (P124),
S56 (P125), S58 (P133),
S72 (P188)
Norton A S22 (7.2), S23 (7.4)
Novak H S64 (P158)
Nowell G S42 (P69)
Nyambo R S26 (P5)
O'Brien M S69 (P178)
O'Brien MER S22 (7.2), S23 (7.4)
O'Flynn KJ S62 (P150), S63 (P151)
O'Hare MJ S49 (P95)
O'Higgins N S14 (3.1), S18 (5.1),
S48 (P94)
O'Higgins NJ S14 (2.6)
O'Neill A S36 (P48)
O'Neill LP S59 (P136)
O'Neill PA S43 (P72), S43 (P74)
O'Reilly DJ S61 (P146)
Oates J S22 (6.8)
Ogunkolade W S19 (5.5)
Oliver RTD S61 (P143), S64 (P156)
Oliver T S25 (P2), S62 (P147),
S66 (P165)
Ong J S62 (P147)
Ord JJ S45 (P81)
Orridge C S11 (1.4)
Oscier D S67 (P169)
Osinsky DS S43 (P75)
Osinsky SP S27 (P9), S43 (P75)
O'Sullivan C S53 (P114)
Ottley C S42 (P69)
Owens C S16 (4.1)
Packham G S15 (3.5), S59 (P135),
S67 (P169)
Padhani A S12 (1.5)
Padhani AR S41 (P66), S44 (P77)
Palit V S30 (P24)
Palmer C S57 (P129), S71 (P183)
Pandha H S16 (3.7), S44 (P76)
Paris AMI S72 (P187)
Parker C S50 (P102)
Parker L S51 (P103)
Parker T S66 (P166)
Parkes A S60 (P142)
Parkins CS S28 (P16)
Parr C S45 (P80), S62 (P149)
Parry A S41 (P68)
Partridge M S23 (7.6)
Patel DB S53 (P112)
Patel KB S12 (1.7)
Patel N S54 (P115), S54 (P118)
Patel PM S47 (P89)
Pathak SK S62 (P148)
Patterson AV S31 (P25)
Patterson LH S33 (P35)
Paulin FEM S34 (P39),
S35 (P42)
Pavillard V S42 (P70)
Payne GS S17 (4.3)
Payne HA S61 (P145)
Peake D S63 (P153)
Pearson ADJ S12 (2.1), S18 (4.7),
S41 (P68), S50 (P101),
S52 (P107), S53 (P111),
S54 (P116)
Pearson G S42 (P69)
Pedley RB S37 (P50), S37 (P51)
Pendlebury S S68 (P174)
Perren T S58 (P132)
Perry PJ S33 (P36)
Petty RD S45 (P82), S48 (P92)
Phelan L S53 (P113)
Phillips E S23 (7.6)
Phillips H S23 (7.3)
Phillips RM S12 (1.7), S28 (P15),
S30 (P24), S31 (P26),
S31 (P27), S32 (P29),
S32 (P30), S42 (P70)
Phillips VA S28 (P15)
Pickering LM S14 (2.7), S25 (P4)
Pinder SE S44 (P78)
Pinkerton CR S17 (4.3), S17 (4.4)
Pledge SD S71 (P185)
Pollard S S69 (P177)
Pollock AM S46 (P84)
Poole CJ S9 (CT1)
Popat S S22 (7.2)
Potter G S27 (P11)
Potter GA S33 (P36)
Powell MEB S24 (7.7)
Powles T S25 (P2), S66 (P165)
Powles TJ S19 (5.3)
Pratt J S57 (P129), S71 (P183)
Price L S41 (P68)
Price RK S49 (P95)
Price T S56 (P125), S58 (P133)
Priest K S22 (7.2)
Prime W S13 (2.4)
Primrose JN S29 (P20)
Pritchard J S16 (4.1)
Pritchard-Jones K S16 (4.2)
Punt J S66 (P166)
Puntis MCA S46 (P83)
Puri R S32 (P29), S32 (P30)
Purohit OP S67 (P170)
Quatan N S16 (3.7)
Quint W S22 (6.7)
Quirke P S21 (6.5)
Radford J S37 (P49)
Radford JA S65 (P161)
Radstone DJ S71 (P185)
Rahman N S16 (4.2)
Raj K S28 (P14)
Ramage J S38 (P56)
Ramalingam S S57 (P130)
Ramesh A S40 (P62)
Rankin EM S33 (P34), S34 (P39),
S35 (P42)
Rao M S56 (P123)
Rao S S56 (P125), S58 (P133)
Rapley L S16 (4.2)
Rapraeger A S13 (2.3)
Raynaud F S21 (6.6)
Redfern CPF S67 (P168)
Reed KA S68 (P174)
Rees R S38 (P56)
Renshaw L S58 (P134)
Rhomberg W S64 (P157),
S64 (P158)
Richards WG S1 (S4)
Richman SD S55 (P119)
Riley P S63 (P153)
Rmali K S46 (P83)
Roberts S S17 (4.6)
Robson H S17 (4.6)
Robson T S32 (P32)
Roggo A S43 (P75)
Ronen S S41 (P67)
Ross P S23 (7.4)
Ross PJ S22 (7.2), S56 (P124),
S56 (P125), S58 (P133),
S72 (P188)
Ross S S11 (1.2)
Rostom AY S20 (5.8)
Ruparelia KC S33 (P36)
Russell N S16 (3.7)
Ryder J S65 (P161)
Rye T S57 (P128), S71 (P186)
Saad S S29 (P17)
Sadozye AH S55 (P121)
Saha V S18 (4.8), S3 (S10),
S54 (P115), S54 (P118)
Sala A S67 (P167)
Salmon R S11 (1.4)
Samuel LM S55 (P121)
Sanders PM S68 (P172)
Sangar V S62 (P150), S63 (P151)
Sangtani BK S72 (P187)
Sanmugathasan A S46 (P85)
Santilli G S67 (P167)
Author Index
S75
British Journal of Cancer (2003) 88(Suppl 1), S73 - S76
& 2003 Cancer Research UK
Sarker D S65 (P159)
Sattar N S61 (P146)
Saunders M S11 (1.3)
Saunders MI S23 (7.3)
Savelyich BSP S24 (7.8)
Schenkein D S65 (P162)
Schey S S28 (P14)
Schmidt-Wolf I S3 (S9)
Schofield AC S26 (P6), S46 (P84),
S59 (P137), S59 (P138)
Schofield J S27 (P10)
Scott DK S52 (P107)
Seargent J S32 (P29)
Seargent JM S64 (P155)
Searle KH S66 (P166)
Seifalian AM S56 (P123)
Senaratne SG S25 (P3)
Sgouros J S68 (P171)
Shaaban AM S13 (2.4), S43 (P74),
S43 (P72)
Shah EF S45 (P80a)
Shah N S11 (1.3)
Shah T S30 (P24), S32 (P29),
S32 (P30)
Shamash J S25 (P2), S61 (P143),
S62 (P147), S66 (P165)
Shanks JH S49 (P97)
Shannon R S16 (4.1)
Sharma RA S62 (P148)
Sharma SK S37 (P50), S37 (P51)
Sharp A S59 (P135)
Shaw EJ S49 (P96)
Shellito P S58 (P133)
Shenoy A S15 (3.6)
Sherlock DJ S38 (P55)
Shnyder SD S27 (P11),
S29 (P19)
Short S S11 (1.3), S41 (P65)
Shringarpure R S65 (P162)
Sibson DR S43 (P74)
Sigan AL S27 (P9)
Singh R S62 (P148)
Singh S S30 (P21)
Sjostrom J S19 (5.4)
Slade R S13 (2.3)
Sludden J S41 (P68)
Smith A S21 (6.5)
Smith AM S35 (P43)
Smith HJ S68 (P173)
Smith IE S22 (7.2)
Smith JL S68 (P174)
Smith KC S43 (P73), S50 (P100)
Smith R S12 (1.5)
Smith RE S34 (P37)
Smith T S15 (3.6), S42 (P71a)
Smyth J S57 (P128)
Smyth JF S13 (2.2), S71 (P186)
Speirs V S20 (5.7), S60 (P142)
Spendlove I S38 (P56), S39 (P57),
S44 (P78)
Spooner D S63 (P153), S9 (CT1)
Sreeja J S58 (P131)
Stanslas J S26 (P8), S29 (P17),
S29 (P18)
Stanway S S53 (P114)
Stein RC S49 (P95)
Stephen J S58 (P131)
Stevens MFG S15 (3.3), S28 (P13),
S29 (P17), S40 (P63)
Stevenson DAJ S59 (P137)
Stevenson F S67 (P169)
Stevenson GT S35 (P43)
Steward WP S60 (P139),
S62 (P148)
Stewart DS S48 (P91)
Stewart KN S46 (P84)
Stewart M S57 (P128), S71 (P186)
Stirling J S12 (1.5)
Stirling JJ S41 (P66)
Stocken D S63 (P153)
Stocken DD S55 (P120)
Stoneham SJ S54 (P115)
Storey DJ S71 (P186)
Stratford I S52 (P109)
Stratford IJ S12 (1.6), S30 (P21)
Stratford MRL S12 (1.7)
Strauss SJ S65 (P162)
Streeter EH S45 (P81)
Streetly M S28 (P14)
Stuart RC S40 (P64)
Stubbs M S41 (P67)
Subar DA S38 (P55)
Suggitt M S31 (P28)
Sumpter K S22 (6.8), S22 (7.2), S23
(7.4)
Sundar S S24 (7.8)
Swaine DJ S42 (P70)
Symonds RP S24 (7.8)
Tait D S20 (6.1)
Talwar D S61 (P146)
Tan HL S33 (P36)
Taylor GA S55 (P122)
Taylor J S12 (1.5), S29 (P19)
Taylor NJ S41 (P66)
Taylor R S16 (4.1)
Tebbutt N S20 (6.1)
Theti D S21 (6.6)
Thies F S26 (P6)
Thomas HD S55 (P122)
Thomas R S50 (P100)
Thomas V S13 (2.5)
Thompson AM S35 (P42)
Thomson GA S34 (P39)
Thornton N S51 (P105)
Thorpe H S21 (6.5)
Thurston DE S29 (P19)
Tierney JF S7 (S28)
Tijani S S64 (P155)
Tilby M S42 (P69)
Tilby MJ S18 (4.7), S39 (P60)
Tisdale MJ S48 (P93), S68 (P172),
S68 (P173)
Titu LV S40 (P62)
Toda T S18 (4.8)
Tolan S S20 (6.2)
Topham C S56 (P124)
Townend SJ S53 (P112)
Townsend PA S59 (P135)
Tozer GM S28 (P16), S30 (P22)
Trott DA S18 (4.7)
Troy H S41 (P67)
Tupper J S30 (P22)
Turnbull A S27 (P12)
Turner AJ S61 (P144)
Turner L S66 (P163)
Tutt A S2 (S6)
Tutt AL S36 (P45)
Tweddle DA S54 (P116)
Twelves C S9 (CT1)
Underwood MA S61 (P146)
Usmani BA S61 (P144)
Vaidya SJ S17 (4.3)
vale C S70 (P180)
Valenti M S21 (6.6)
Valkovskaya NV S27 (P9)
van Delft F S54 (P118)
van Delft FW S54 (P115)
van der Zee A S40 (P64)
Veal GJ S41 (P68), S53 (P111), S67
(P168)
Vernon DI S27 (P10)
Vinjamuri S S15 (3.6)
Volpato M S31 (P26)
von Zglinicki T S39 (P60)
Vousden K S7 (S25)
Vujanic M S16 (4.1)
Walker C S15 (3.6), S42 (P71a),
S49 (P96)
Walker D S66 (P166)
Walker J S16 (4.1), S21 (6.5)
Walkington J S58 (P134)
Wallace HM S59 (P137)
Walshe C S36 (P45)
Wang Q S50 (P102)
Ward C S35 (P42), S56 (P125)
Ward CY S33 (P34)
Wardman P S27 (P9), S30 (P23)
Wardrop J S34 (P39), S35 (P42)
Warnke PC S15 (3.6), S42 (P71a),
S49 (P96)
Watkins D S58 (P133)
Watkins G S45 (P80), S60 (P141)
Watson SA S49 (P98)
Waxman J S4 (S16)
Webb D S10 (CT5)
Weeden S S9 (CT3), S70 (P179)
Wells C S19 (5.6)
Westwell AD S28 (P13)
Wheatley K S10 (CT5)
Wheelhouse RT S28 (P15)
Wheelwright R S70 (P182)
Whelan J S16 (3.7), S53 (P114),
S44 (P76)
Whelan JS S17 (4.4)
Whelan M S44 (P76)
Whelan MA S16 (3.7)
Whiting G S70 (P181)
Whitworth MK S13 (2.3)
Wilkie ED S22 (7.1)
Wilkinson GP S29 (P19)
Williams AR S57 (P128),
S71 (P186)
Williams FJ S23 (7.3)
Williams M S65 (P159)
Williamson AJK S52 (P110)
Willner S S57 (P130)
Wilsher NE S33 (P36)
Wilson EM S23 (7.3)
Wilson G S13 (2.3)
Wilson L S11 (1.2), S63 (P152)
Wilson P S62 (P147)
Wilson PD S61 (P143)
Wilson WR S31 (P25)
Winslet MC S22 (6.7),
S56 (P123)
Wisman BA S40 (P64)
Wolf CR S35 (P42)
Wolf RC S33 (P34)
Wood KM S54 (P116)
Woodcock M S11 (1.1)
Workman P S41 (P67)
Wotherspoon A S20 (6.1)
Wushishi F S16 (3.7)
Wyke SM S68 (P173)
Yahaya RNR S29 (P18)
Yakkundi A S32 (P32)
Yang W S56 (P123)
Yong SM S46 (P84)
Young BD S18 (4.8)
Young L S14 (3.1), S18 (5.1),
S27 (P12)
Young LS S14 (2.6)
Zalutsky MR S6 (S22)
Zhao J S39 (P58)
Zivanovic M S38 (P53)
Author Index
S76
British Journal of Cancer (2003) 88(Suppl 1), S73 - S76 & 2003 Cancer Research UK

				

